# Acute Compartment Syndrome Following Repeated Calf Kicks in an Elite-Level Kickboxing Athlete: A Case Report of a Rare Non-Fracture-Related Complication

**DOI:** 10.3390/jfmk11020206

**Published:** 2026-05-23

**Authors:** Sacha Beca, Bonnange-Michael Fundu Ngoie Zola, Kalenga Gracia Bundo, Arnaud Delafontaine, Virginie Cordemans

**Affiliations:** 1 Free University of Brussels (ULB), CP 619-1070 Brussels, Belgium; sacha.beca@ulb.be; 2 Faculty of Medicine, University Hospital Center-Brugmann, CHU Brugmann, 1020 Brussels, Belgium; bonnange-michael.fundungoiezola@chu-brugmann.be (B.-M.F.N.Z.); kalenga.bundo@chu-brugmann.be (K.G.B.); 3 CIAMS, Paris-Saclay University, 91404 Orsay, France; 4 Laboratoire D’Anatomie Fonctionnelle, Faculté des Sciences de la Motricité, Université Libre de Bruxelles, Route de Lennik 808, CP 619-1070 Brussels, Belgium; 5 Department of Orthopedics and Traumatology, University Hospital Center-Brugmann, CHU Brugmann, 1020 Brussels, Belgium; virginie.cordemans@chu-brugmann.be

**Keywords:** lower-leg injury, combat sports, posterior compartment syndrome, intramuscular hematoma, emergency fasciotomy, repetitive blunt trauma

## Abstract

**Background:** Acute compartment syndrome (ACS) is a limb-threatening surgical emergency most commonly associated with fractures or high-energy trauma. Non-fracture-related ACS in athletes is uncommon and may lead to delayed diagnosis. Repetitive blunt trauma during combat sports has rarely been described as a potential mechanism. **Case Methods:** The case concerns a 21-year-old elite-level kickboxing athlete who developed acute compartment syndrome of the left lower leg following repeated calf kicks sustained during sparring. The patient presented with rapidly progressive calf pain, swelling, compartment firmness, paresthesias and weight bearing difficulties. CT angiography demonstrated diffuse edema of the posterior compartments associated with a large intramuscular soleus hematoma without active arterial bleeding. **Results:** In view of the severity of the symptoms and the characteristic clinical presentation, an emergency fasciotomy was performed in operating room. Progressive closure was achieved using the vessel loop shoelace technique, allowing gradual tension-free closure. Wound healing progressed without infection, and physiotherapy was introduced with joint mobilization. The patient achieved full functional recovery after 6 months. **Conclusions:** This case illustrates an atypical etiology of ACS—repetitive targeted calf strikes—and underscores the importance of early recognition even in the absence of fracture or major trauma. Clinical vigilance remains paramount, and prompt surgical intervention is critical to prevent irreversible muscle and nerve damage. Awareness of such mechanisms is particularly relevant for clinicians managing athletes in combat sports. To our knowledge, this is the first documented case of ACS secondary to repeated calf kicks in kickboxing.

## 1. Introduction

### 1.1. Compartment Syndrome

Acute compartment syndrome (ACS) is a serious limb-threatening surgical emergency characterized by increased pressure within a closed osteofascial compartment, resulting in impaired tissue perfusion and progressive ischemic injury [1]. First described more than a century ago, ACS remains one of the most feared orthopedic emergencies in both trauma and sports medicine because delayed treatment may lead to irreversible muscle necrosis, neurological damage, limb dysfunction, or amputation [2].

The pathophysiology involves a progressive rise in intracompartmental pressure, impairing venous outflow and microcirculation, ultimately compromising tissue oxygenation once perfusion pressure falls below a critical threshold [3,4].

Diagnosis is primarily clinical and relies on rapidly progressive pain disproportionate to the apparent injury, pain exacerbated by passive stretching, tense compartment swelling, and neurological symptoms such as paresthesia. On the other hand, clinical examination alone may lack sensitivity, and manual detection of compartment firmness has been shown to be unreliable [5]. Although the classic “5 Ps” (pain, pallor, paresthesia, paralysis, and pulselessness) are frequently described, their sensitivity remains limited, particularly during the early stages of ACS [6].

Intracompartmental pressure measurement may support diagnosis in equivocal cases; however, urgent surgical decompression should not be delayed when clinical suspicion is high. Nevertheless, the diagnosis of ACS remains challenging, with significant interobserver variability and no universally reliable diagnostic test currently available [7,8,9]. Although biomarkers such as creatine kinase (CK), lactate, and other serum parameters have been investigated as adjunctive markers, routine laboratory tests are not considered primary diagnostic tools [10].

Emergency fasciotomy remains the gold-standard treatment and should be performed promptly to restore tissue perfusion and preserve neuromuscular function [11].

ACS most commonly occurs following fractures, high-energy trauma, vascular injury, or severe soft tissue damage, particularly involving the tibia and lower leg compartments [12]. However, non-fracture-related ACS has increasingly been described in athletes following exertion, blunt trauma, muscle tears, or repetitive microtrauma. Sports-related ACS has been reported in running, soccer, American football, roller sports, and combat sports, although these presentations remain relatively uncommon and may contribute to delayed diagnosis because of their atypical and often low-energy presentation [13].

In 1975, Rorabeck and Macnab [14] explained in their study that exercise or extreme exertion leads to an increase in compartment contents through a combination of acute muscular hypertrophy and fluid accumulation. They found that muscles can increase their volume by up to 20% during exercise. This is associated with electrolyte shifts and possibly hemorrhage from ruptured muscle fibers, leading to increased pressure within a fixed compartment space and initiating a cycle of relative muscle ischemia, edema, rising intracompartmental pressure, reduced arterial perfusion, and further muscle ischemia.

In their study, Lamplot et al. [15] reported 22 cases of lower-extremity compartment syndrome of the leg among NFL players over an 18-year period. All cases presented after direct impact during regular-season games; however, only two cases were associated with fractures. In 2024, Bukhamas et al. [16] described the case of a 19-year-old male MMA athlete who developed acute compartment syndrome (ACS) of the thigh following a direct kick during a fight, without fracture. They concluded that thigh ACS is an uncommon condition, and that its diagnosis may be delayed or missed, particularly in the sporting context, due to its insidious progression. Baumfeld et al. [17] described the case of a 16-year-old professional soccer player who developed ACS after 90 min of intense practice, without trauma or symptoms during the session. They also concluded that non-traumatic ACS after physical activity is a rare clinical entity.

### 1.2. Kickboxing

Kickboxing is a combat sport combining punches, kicks, and knee strikes delivered at high speed and force [18]. As in other striking combat sports, soft-tissue injuries, contusions, hematomas, and lacerations represent the most frequently reported injuries. Lower extremity injuries are the most commonly reported, while head, face, and neck injuries also account for a substantial proportion of injuries in kickboxing and Muay Thai [19].

**Figure 1:**
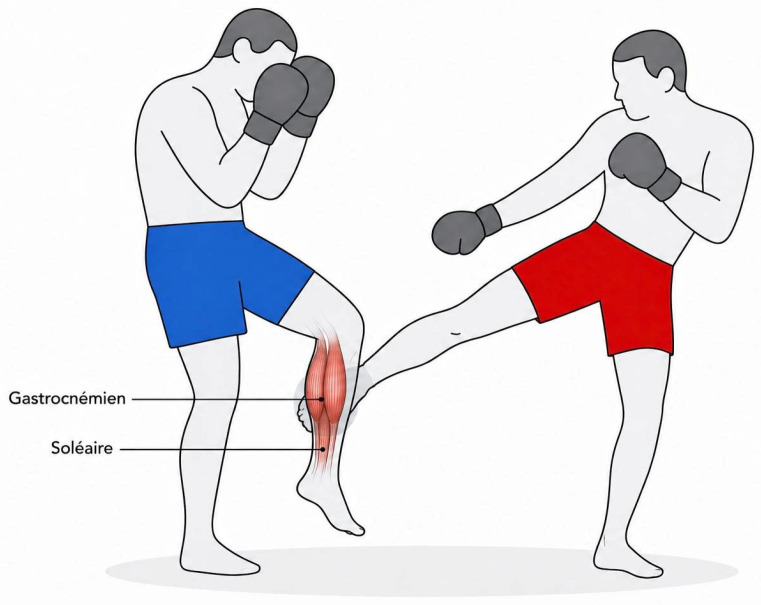
Schematic representation of repeated calf kicks targeting the posterior aspect of the lower leg (gastrocnemius and soleus muscles) in kickboxing.

Among these techniques, calf kicks specifically target the posterior aspect of the lower leg to impair balance, mobility, and weight-bearing capacity by inducing progressive pain and muscular dysfunction (Figure 1). Repetitive impacts to the calf may generate cumulative blunt microtrauma, intramuscular edema, hematoma formation, and increased compartment pressure, particularly within the relatively noncompliant deep posterior compartment. Despite the growing popularity of calf kicks in modern kickboxing and mixed martial arts (MMA), ACS secondary to repetitive calf-targeted trauma has rarely been reported in the literature [16,19,20,21,22,23].

The case concerns a 21-year-old elite-level kickboxing athlete who developed acute compartment syndrome of the lower leg following repeated calf kicks sustained during sparring. This case highlights the diagnostic challenges of non-fracture-related ACS in combat sports and emphasizes the importance of early clinical recognition and urgent surgical management.

## 2. Case Presentation

A 21-year-old Caucasian male athlete (175 cm, 80 kg), who had been practicing kickboxing seriously for several years and competing at a professional level, presented to the emergency department in the evening with progressively worsening severe pain in the left calf that had developed in the early afternoon the same day during a kickboxing sparring session against a veteran opponent. The session involved repeated forceful “calf kicks,” consisting of direct strikes to the posterior aspect of the lower leg. The pain, initially moderate, rapidly became intolerable and was associated with swelling, localized induration, and marked functional impairment limiting lower-limb mobility and weight bearing. The patient also reported paresthesia in the left leg, although no objective sensory or motor deficit was identified on examination.

Clinical assessment demonstrated marked swelling and palpable firmness predominantly involving the posteromedial aspect of the calf, consistent with deep posterior compartment involvement. Passive ankle movements and stretching of the posterior compartment muscles provoked intense pain associated with tense “wood-like” calf consistency, involving both the anterior and posterior aspects of the leg, making findings highly suggestive of acute compartment syndrome. Distal pulses were palpable, and capillary refill remained preserved. The patient had no relevant medical, surgical, or allergic history.

Laboratory investigations performed in the emergency department demonstrated elevated lactate dehydrogenase (LDH) at 308 U/L (reference range: 135–225 U/L) and creatine kinase (CK) at 554 U/L (reference range: 39–308 U/L). The remainder of the blood work was unremarkable, including hemoglobin at 15 g/dL, normal renal function, a normal electrolyte panel, and normal liver enzymes.

**Figure 2:**
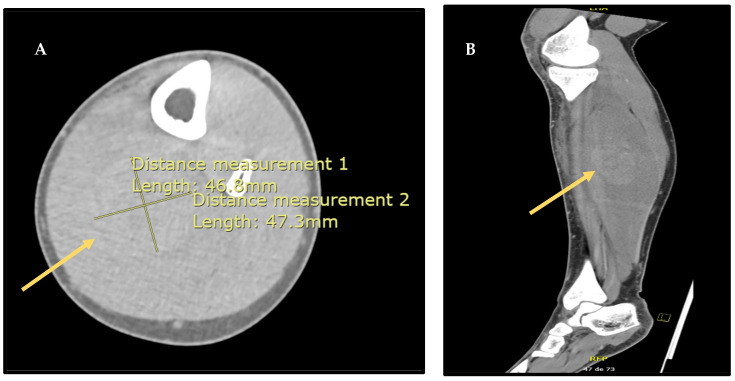
Imaging (axial (**A**) and sagittal (**B**) views) revealed diffuse edema of the left leg with pronounced swelling predominantly involving the deep posterior compartment. The yellow arrows indicate the hematoma. A well-circumscribed intramuscular hematoma measuring approximately 4.5 × 4.5 cm was identified within the left soleus muscle. Following intravenous contrast administration, there was no evidence of active arterial bleeding or contrast extravasation. Mild focal compression of the posterior tibial artery was observed at its mid-portion, while arterial flow and distal vessel patency remained preserved.

Given the progressive onset of symptoms following relatively minor trauma, with no clinical signs of bone lesion, laxity, or articular instability, CT angiography was performed because of the atypical mechanism of injury and concern for an underlying vascular lesion or active bleeding source. Imaging demonstrated diffuse edema predominantly involving the deep posterior compartment of the left leg, associated with a large intramuscular hematoma within the left soleus muscle measuring approximately 4.5 × 4.5 cm (Figure 2). No active arterial extravasation was identified.

Persistent pain despite non-opioid and weak opioid analgesics, combined with the imaging findings, strongly supported the diagnosis of acute compartment syndrome induced by repetitive blunt calf trauma. Given the highly suggestive clinical presentation and rapidly progressive symptoms, intracompartmental pressure measurements were not obtained in order to avoid delaying emergent surgical decompression. The patient was urgently referred to the orthopedic surgery team and received intravenous analgesia prior to surgery.

CT imaging demonstrated a predominant hematoma within the deep posterior compartment, consistent with the clinical findings of diffuse calf tension, marked firmness on palpation, and severe pain elicited by passive ankle mobilization. Given the diffuse compartment tension and the risk of progression to multicompartment involvement, a four-compartment fasciotomy was deemed necessary and was performed under general anesthesia using medial and lateral incisions.

**Figure 3:**
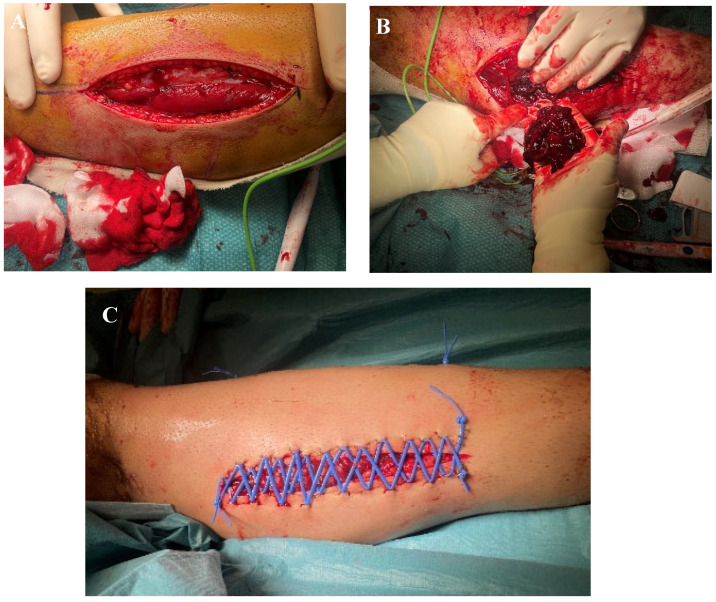
Illustrates the intraoperative management: (**A**) Lateral incision used for decompression of the anterior and lateral compartments. (**B**) Medial incision used for decompression of the posterior compartments. (**C**) Shoelace technique.

The procedure included decompression of all four compartments, debridement of the swollen soleus muscle within the deep posterior compartment (Figure 3A), and partial evacuation of the deep hematoma Figure 3B). The muscles appeared well vascularized and demonstrated a brisk contractile response to electrical stimulation, with no evidence of muscle necrosis or active bleeding. Temporary closure was achieved using the vessel loop shoelace technique to minimize tension and facilitate delayed wound closure (Figure 3C).

Postoperative management included analgesia without nonsteroidal anti-inflammatory drugs, administration of Exacyl 1 gr 3 times per day for four days, withholding of anticoagulation, and close daily clinical monitoring.

A second-look surgery was performed less than 24 h later to evacuate a residual deep hematoma located between the gastrocnemius and soleus muscles, debride the muscular tear, and apply hemoclips to control persistent bleeding following hematoma evacuation. The fasciotomy sites were inspected, thoroughly irrigated, and again temporarily closed using the vessel loop shoelace technique. Postoperative care included strict rest, regular cold application, mobilization with strict non-weight-bearing precautions, and avoidance of active loading of the lower limb. During the postoperative course, the patient developed severe anemia requiring transfusion of two units of packed red blood cells.

**Figure 4:**
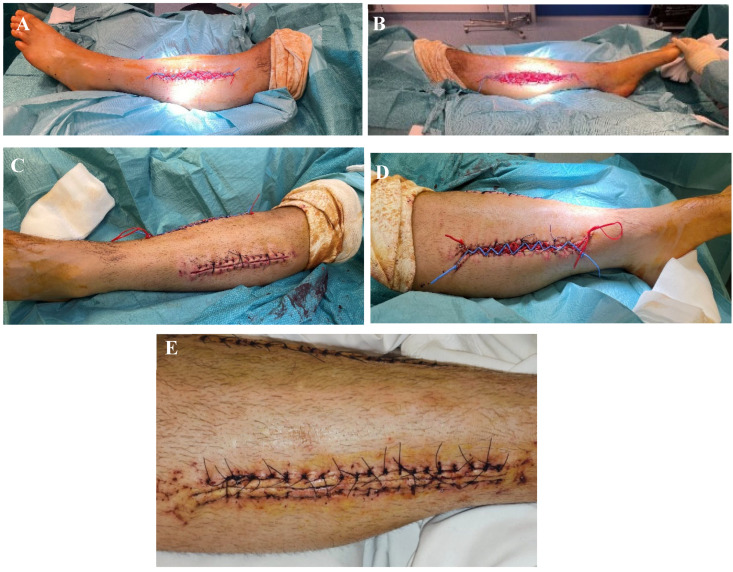
Panels (**A**,**B**) illustrate medial and lateral shoelace closure on postoperative day 7, respectively. Panels (**C**,**D**) illustrate definitive lateral closure and persistent medial shoelace closure on postoperative day 10. (**E**) illustrates definitive medial closure on postoperative day 14.

Between postoperative days 7 and 14, multiple procedures were performed for progressive wound management, including limited debridement of superficial devitalized tissue, refreshing of the wound edges, and gradual approximation of the fasciotomy wounds through sequential tightening of the vessel loops. Definitive cutaneous closure was achieved on postoperative day 14 following removal of the temporary closure devices. Figure 4 illustrates the different stages of wound closure.

Pharmacological thromboprophylaxis was initiated after definitive wound closure once the bleeding risk was considered controlled. Pain progressively improved, and gentle joint mobilization at bed was initiated while maintaining offloading with crutches. The patient was discharged from the hospital 15 days after the initial emergency surgery.

The first follow-up evaluation occurred one week after hospital discharge. Localized redness without fever or systemic signs of infection prompted close monitoring and specialist reassessment. Subsequent follow-up examinations demonstrated progressive wound healing without evidence of infection, gradual resumption of weight-bearing, sequential suture removal, and initiation of physiotherapy.

**Figure 5:**
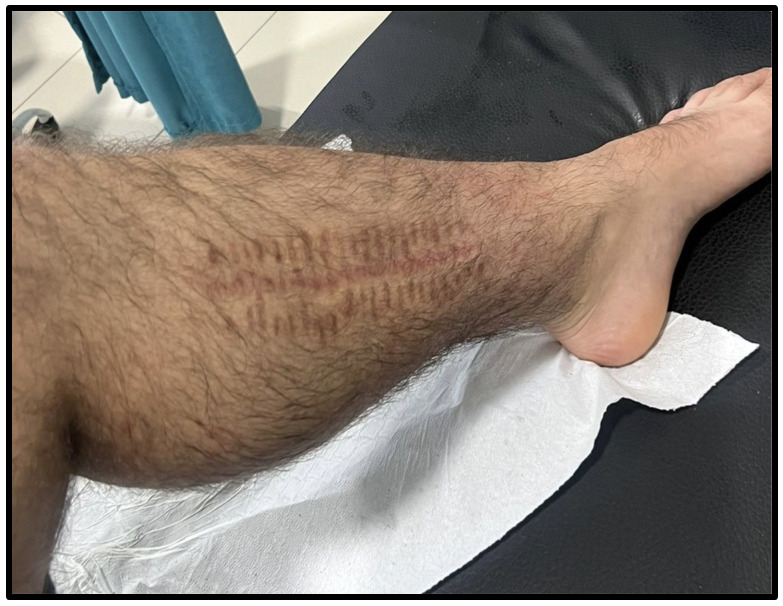
Clinical appearance six months after surgery demonstrating complete wound healing following fasciotomy.

By the three-month postoperative follow-up, the fasciotomy wounds had completely healed, with full restoration of skin integrity and no residual inflammatory or infectious complications (Figure 5). Progressive recovery of calf strength and ankle mobility was observed, and no postoperative neurological deficit was identified during follow-up examinations. Return to contact sports was authorized following complete wound healing, painless full weight-bearing, restoration of full ankle range of motion, symmetric calf strength compared with the contralateral side, and absence of neurological deficits. Six months after surgery, the patient had fully resumed contact sports, including kickboxing, without pain, swelling, or functional limitation following recovery from predominantly deep posterior acute compartment syndrome.

The patient’s clinical course and surgical management are summarized in two tables. Table 1 outlines the chronological timeline of surgical procedures and postoperative care, while Table 2 summarizes the surgical interventions and associated postoperative management.

## 3. Discussion

This case illustrates a rare but severe presentation of acute compartment syndrome (ACS) of the posterior compartment of the lower leg, occurring after repeated calf kicks during a kickboxing training session.

While ACS most commonly follows tibial fractures or high-energy trauma, several cases have been reported without bony injury, occurring secondary to repetitive microtrauma or compressive intramuscular hematoma formation. In this case, repeated strikes to the posterior aspect of the leg likely caused soleus muscle injury with subsequent deep hematoma formation, progressively increasing intracompartmental pressure. This atypical mechanism—unrelated to fracture—carries a risk of delayed diagnosis, emphasizing the importance of recognizing unusual presentations of ACS in striking sports [19,20]. Previous reports have described non-fracture ACS following blunt sports-related trauma, including soccer, rugby, and martial arts injuries; however, ACS specifically associated with repetitive calf kicks in kickboxing remains exceptionally uncommon.

Several cases of sports-related ACS without major fracture have previously been reported, including exercise-induced ACS of the lower leg, low-energy sports trauma, and ACS occurring in combat-sport athletes or mixed martial arts fighters [16,21,22,23]. These observations highlight the diagnostic challenges of ACS in atypical low-energy mechanisms and support the importance of early recognition in athletic populations.

Esmail et al. [22] reported the case of a 17-year-old boy with increasing leg pain occurring 4 days after repetitive place-kicking practice with his right leg during a school football camp. The diagnosis was established after 4 days of evolution. They concluded that exercise-induced compartment syndrome may be initially missed due to its similarity to an ankle sprain. Another case report highlighting a thigh ACS in an MMA athlete from Bukhamas et al. [16] concluded that, in the absence of an obvious history of trauma and severe pain, the diagnosis of thigh ACS can be easily missed.

Calf kicks are a striking technique targeting the calf muscles, and their repetition can, in rare cases, cause deep muscle contusions. In the present case, this repetitive mechanism resulted in the formation of a compressive hematoma, leading to acute compartment syndrome. To our knowledge, no similar case specifically related to repeated calf kicks in kickboxing has been previously described, underlining the need to consider such mechanisms in kickboxing and, more broadly, in sports medicine.

The diagnosis of ACS remains primarily clinical [24]. In our case, the patient presented with the classic features: disproportionate pain, swelling, muscular firmness, paresthesia, and pain exacerbated by passive stretching. CT angiography demonstrated a deep intramuscular hematoma without active bleeding, supporting the clinical suspicion of ACS. Although intracompartmental pressure measurement can assist in diagnosis, it was not performed here due to the clear clinical presentation and the urgent need for surgical decompression. This management aligns with current recommendations, which emphasize not delaying fasciotomy when ACS is clinically evident [24].

Furthermore, laboratory investigations performed at admission demonstrated elevated lactate dehydrogenase (LDH) and creatine kinase (CK) levels, reflecting underlying muscle injury but remaining well below the markedly elevated values typically associated with late-stage ACS and extensive rhabdomyolysis [25]. Although several studies have explored the diagnostic utility of serum biomarkers in ACS, these parameters lack sufficient sensitivity to reliably confirm or exclude the diagnosis [10,26]. Their primary clinical value lies in assessing the extent of muscle injury and monitoring for complications such as rhabdomyolysis rather than guiding surgical decision-making. In the present case, the relatively moderate biological abnormalities despite severe clinical findings likely reflect early diagnosis and prompt surgical intervention before the development of irreversible ischemic muscle necrosis [26]. Consequently, a “normal” or moderately elevated biochemical profile cannot reliably exclude ACS. In this case, the discrepancy between severe clinical presentation and biological profile simply confirms that the patient was treated in the pre-necrotic ischemic window.

Management of ACS relies on emergency fasciotomy through medial and lateral approaches, allowing decompression of all four compartments and restoration of tissue perfusion [27]. In this case, fasciotomy was performed in two stages, with evacuation of the hematoma and complete decompression of the compartments. Delayed closure was achieved using the vessel loop shoelace technique, allowing progressive wound approximation without tension and avoiding the need for skin grafting. This method, described in the literature as simple, cost-effective, and efficient, promotes wound healing, reduces the risk of infection, and yields superior cosmetic outcomes [28]. In our patient, this approach facilitated rapid recovery and complete functional restoration at 6-month follow-up.

From a sports medicine perspective, this case highlights that repeated calf kicks, although commonly perceived as relatively benign in combat sports, may rarely result in severe soft-tissue injury and acute compartment syndrome even in the absence of fracture. Persistent disproportionate pain, progressive swelling, pain on passive stretching, or neurological symptoms following repetitive blunt calf trauma should prompt urgent evaluation and early surgical referral in combat-sport athletes.

This case carries several limitations inherent to the nature of a single case report. The isolated nature of this observation limits the generalizability of findings to other combat sports practitioners. The absence of intracompartmental pressure measurement before fasciotomy constitutes a limitation, although the clinical and imaging evidence was highly suggestive. The causal relationship between calf kicks and the development of ACS remains hypothetical, albeit supported by a coherent clinical sequence. Indeed, the patient was not able to provide details regarding the exact number of strikes, training phase, sparring intensity, weight category, level of competition, session objectives, or precise competitive background. We assume that the emergency context and the language barrier may explain this lack of information.

At final follow-up, the patient demonstrated full ankle range of motion, normal gait, painless full weight-bearing, and symmetric calf strength compared with the contralateral side. Because the patient lived abroad and returned to his home country after recovery, longer-term follow-up was not feasible, limiting assessment of potential late sequelae or recurrence.

## 4. Conclusions

This case illustrates a rare but severe presentation of acute compartment syndrome following repeated calf kicks in kickboxing. It emphasizes the importance for clinicians of recognizing this potential complication, even in the absence of fracture or major trauma.

Although imaging demonstrated a localized hematoma within the deep posterior compartment, the decision to perform a complete multi-compartment fasciotomy was guided primarily by the global clinical findings, including diffuse compartment tension and severe pain elicited by passive stretching. This underscores the primacy of clinical examination over imaging in the diagnosis and management of acute compartment syndrome. Prompt surgical intervention is essential to prevent irreversible ischemic and neurological sequelae. Finally, this observation highlights the importance of increasing awareness among athletes, coaches, and medical teams regarding the potential risk of acute compartment syndrome associated with repetitive calf-targeted blunt trauma in combat sports.

## Figures and Tables

**Table 1:** Timeline of surgical procedures and postoperative care.

Date	Timeline	Procedure/Care	Key Details
19 February 2025	Day 1 (evening)	Symptom onset and acute compartment syndrome	Acute calf pain following kickboxing sparring, associated with swelling and paresthesias
20 February 2025	Day 1 (night)	Emergency fasciotomy of the left leg	Medial and lateral incisions, hematoma evacuation, soleus debridement, and temporary closure using vessel loops
21 February 2025	Day 2 (in the morning)	Second-look surgery	Evacuation of residual hematoma, placement of hemoclips, repeat temporary closure using the vessel loop shoelace technique
24 February 2025	Day 5	Blood transfusion	Severe postoperative anemia (Hb 6.2 g/dL) requiring transfusion of two units of packed red blood cells
28 February 2025	Day 9	Complex wound management	Debridement, wound irrigation, progressive reduction in wound openings, and tightening of vessel loops
3 March 2025	Day 12	Progressive wound closure	Additional debridement, progressive tightening of the shoelace closure system
5 March 2025	Day 14	Definitive wound closure	Donati sutures, initiation of passive mobilization, introduction of prophylactic anticoagulation
13 March 2025	Day 22	First postoperative follow-up	Clean wounds with mild localized redness without fever; infectious disease consultation requested
20 March 2025	Day 29	Second postoperative follow-up	Removal of external sutures, initiation of progressive mobilization, and physiotherapy planned
27 March 2025	Day 36	Third postoperative follow-up	Improved weight-bearing capacity, clean wounds, and removal of remaining internal sutures

**Table 2:** Summary of surgical interventions and postoperative management.

Date	Timeline	Intervention	Main Description	Key Postoperative Care
20 February 2025	Day 1	Emergency left leg fasciotomy	Medial and lateral fasciotomies with evacuation of the soleus hematoma, muscle debridement, and temporary closure using vessel loops	Analgesia without NSAIDs, withholding prophylactic anticoagulation for 48 h, daily wound care, limb elevation, and avoidance of compression
21 February 2025	Day 2	Second-look surgery and hematoma evacuation	Removal of vessel loops, evacuation of the residual hematoma located between the gastrocnemius and soleus muscles, hemostasis using hemoclips, extensive irrigation, and repeat temporary closure using vessel loops	Analgesia, strict rest, leg elevation, regular cold application, and avoidance of physiotherapy or upright mobilization
28 February 2025	Day 9	Complex wound management and progressive closure	Debridement, gentle refreshing of wound edges, irrigation, evacuation of liquefied hematoma, and progressive reduction in wound openings by sequential tightening of vessel loops	Daily local wound care, non-compressive dressings, and discontinuation of prolonged antibiotic therapy
3 March 2025	Day 12	Progressive wound closure	Removal of staples and vessel loops, wound-edge debridement, partial closure using Vicryl and Donati sutures, and additional progressive tightening of the closure system	Analgesia, twice-daily local wound care, cold application, leg elevation, and no prophylactic anticoagulation
5 March 2025	Day 14	Definitive wound closure	Removal of temporary closure devices, wound irrigation, and definitive closure using Donati sutures	Initiation of joint mobilization, non-weight-bearing with crutches, introduction of prophylactic anticoagulation, and discharge planning

## Data Availability

All data supporting the findings of this case report are included in the manuscript or are available from the corresponding author upon reasonable request.
